# An equivalency and efficiency study for one year digital pathology for clinical routine diagnostics in an accredited tertiary academic center

**DOI:** 10.1007/s00428-025-04043-3

**Published:** 2025-02-18

**Authors:** Viola Iwuajoku, Kübra Ekici, Anette Haas, Mohammed Zaid Khan, Azar Kazemi, Atsuko Kasajima, Claire Delbridge, Alexander Muckenhuber, Elisa Schmoeckel, Fabian Stögbauer, Christine Bollwein, Kristina Schwamborn, Katja Steiger, Carolin Mogler, Peter J. Schüffler

**Affiliations:** 1 Institute of Pathology, Technical University of Munich, Munich, Germany; 2 Munich Data Science Institute (MDSI), Munich, Germany; 3 Munich Center for Machine Learning (MCML), Munich, Germany

**Keywords:** Digital pathology, Digital transition, Validation study, Efficiency

## Abstract

Digital pathology is revolutionizing clinical diagnostics by offering enhanced efficiency, accuracy, and accessibility of pathological examinations. This study explores the implementation and validation of digital pathology in a large tertiary academic center, focusing on its gradual integration and transition into routine clinical diagnostics. In a comprehensive validation process over a 6-month period, we compared sign-out of digital and physical glass slides of a wide range of different tissue specimens and histopathological diagnoses. Key metrics such as diagnostic concordance and user satisfaction were assessed by involving the pathologists in a validation training and study phase. We measured turnaround times before and after transitioning to digital pathology to assess the impact on overall efficiency. Our results demonstrate a 99% concordance between the analog and digital reports while at the same time reducing the time to sign out a case by almost a minute, suggesting potential long-term efficiency gains. Our digital transition positively impacted our pathology workflow: Pathologists reported increased flexibility and satisfaction due to the ease of accessing and sharing digital slides. However, challenges were identified, including technical issues related to image quality and system integration. Lessons learned from this study emphasize the importance of robust training programs, adequate IT support, and ongoing evaluation to ensure successful integration. This validation study confirms that digital pathology is a viable and beneficial tool for accurate clinical routine diagnostics in large academic centers, offering insights for other institutions considering similar endeavors.

## Introduction

The transition of routine histopathological laboratories into the digital realm is a pivotal and crucial phase in the broader digital transformation of pathology. During the last decades, optical pathology has gradually changed with the introduction of digital cameras capturing still images or allowing for live examination of slides (dynamic images) [1]. This change opens a myriad of possibilities [2], including access to extensive datasets for artificial intelligence (AI)-driven analyses [3, 4]; the facilitation of remote work setups for medical professionals [5–7]; and expedited sharing of images and data for research publications, conferences, and tumor boards. In many scenarios, the new digital tools save time and money. For example, digital image-sharing systems across medical institutions can significantly reduce healthcare costs by reducing the need for additional slides and duplicate tests for patients transferring to a different hospital [8, 9]. However, embracing a fully digital workflow entails substantial efforts in both technical and personnel domains. The first requires a carefully planned installation of digital scanning hardware; an image management and storage system; and their integration into the existing infrastructure, the laboratory workflow, and the laboratory information system. Second, it necessitates a well-conceived and adaptable change management to minimize friction, especially in the personnel sector, ensuring that the valuable potential of talented yet possibly change-resistant employees is not forfeited. Finally, adequate validation of the new technology is imperative when it comes to patient sample management and analysis. Although many laboratories have reported a successful transition to digital pathology (DP) [10–16], such as an increasing number of cases diagnosed per year [5, 17–21], with accumulated evidence of comparable concordance between DP-based and conventional microscopic diagnosis in various sample types [22], digital slides can rapidly and readily be transferred between sites, second opinions can be acquired, and they may be used to swiftly and simply find prior or archived cases [23], providing guidelines [24, 25], and experiences [23, 26], DP is still a niche technology requiring considerable investments in time, money, and energy. The transition strategies are many folds and can be rapid or gradual, e.g., a hospital in Caltagiro Italy, implemented and transitioned to a fully digital workflow in 4 months [29]. The COVID-19 pandemic did force many pathology labs to transition to DP [27]. These examples underscore the diverse strategies and timelines involved in deploying DP, highlighting the need for adaptable and scalable implementation models. There remains a need to align the transition to DP with national and international guidelines, ensuring efficiency, reliability, and scalability. Further, the effect of DP on case review efficiency is still underexplored in current studies.

This article encapsulates the digital transition of the pathology department of our tertiary academic center accredited by the National Accreditation Body of Germany (DAkkS) according to DIN EN ISO/IEC 17020, detailing the technical and personnel challenges encountered, the extent of national and international guidelines influencing the success and scalability of DP implementation, the extensive validation strategy, and digital workflow efficiency of case sign-out times. Additionally, it offers a comprehensive exploration of potentially problematic interfaces in the shift of routine operations to a digitalized workflow and enables pathologists the opportunity to expand their expertise on a global scale [28].

## Transition from retrospective to prospective scanning

**Fig. 1:**
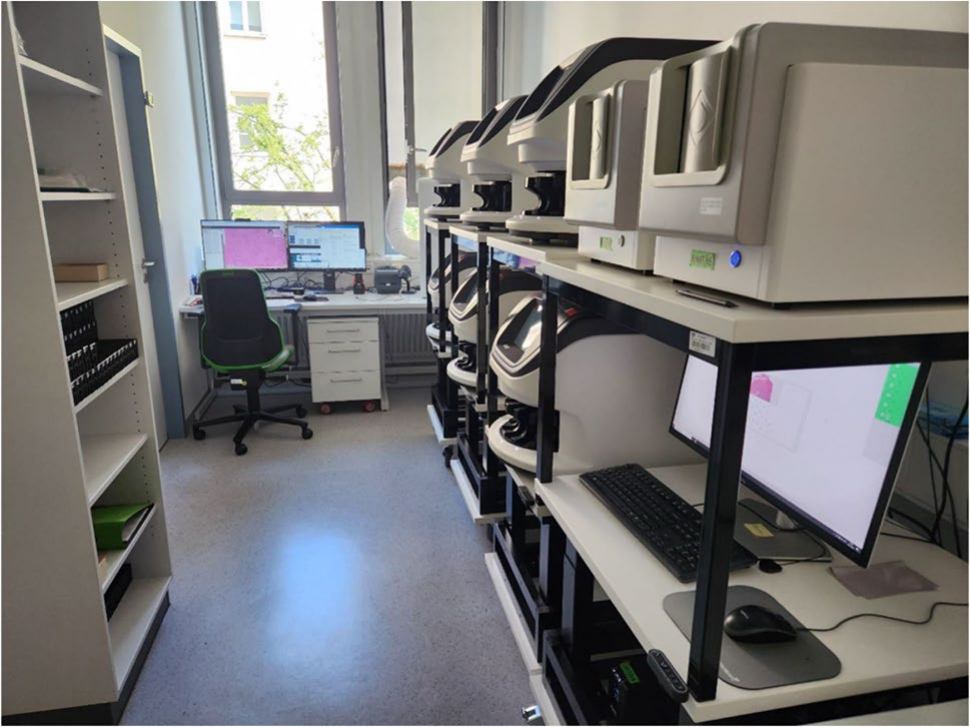
Our current scanning room with six Aperio GT450 Dx scanners and two Objective Imaging GL20 Scanners

Our selection criteria for a slide scanner were image quality, scanning speed, loading capacity, compatibility with stainer racks, our Laboratory Information System (LIS, Nexus Pathology (Nexus AG)) and double-width slides, diagnostic usability, continuous loading ability, scanner size, intuitive and easy user interface, and open file format for research. After testing several models, we started our DP implementation in 2021 using two Aperio GT450Dx (Leica Biosystems) scanners, retrospectively scanning archive slides to acquire proficiency with the overall workflow and IT infrastructure without impacting routine diagnostics. During this time, an interface between scanners and our Laboratory Information System (LIS, Nexus Pathology (Nexus AGLIS) was established, and storage and additional scanners were successively acquired. Due to space limitations in the main laboratory, we accommodate a dedicated scanning room with customized lifting tables, each fitting two scanners vertically (Fig. 1).

After collecting first experiences in the first 2 years, we continuously *transitioned to prospective scanning* [23]. For this, we prospectively scanned subgroups of cases, starting with neuropathology, then immunohistochemistry, then biopsies, and so forth. By this successive approach, we could steadily validate and increase our scanning capacity if needed. This transition period was in a hybrid mode, meaning that glass slides would still be delivered after scanning to the pathologists offering both digital and physical tissue slides. In biweekly DP meetings, a team of representatives from the lab, digital lab, and pathologists evaluated and adjusted the workflow if needed. Adjustments included optimized tissue preparation (tissue fragments placed closer together and more centric on the slide during embedding to avoid long scanning time), and the prioritization of biopsies during embedding as the scanners can process them quicker [23]. An important consideration was the laboratory turnaround time (TAT) and the delay of slide delivery: Even if individual slides are quickly scanned within 1–3 min, a rack of 20 slides requires 40–50 min to be finished, depending on the amount of tissue. To reduce this *scanning delay*, we increased the scanning capacity with more scanners and prioritized biopsy cases on a day over larger resection specimens [23].

**Fig. 2:**
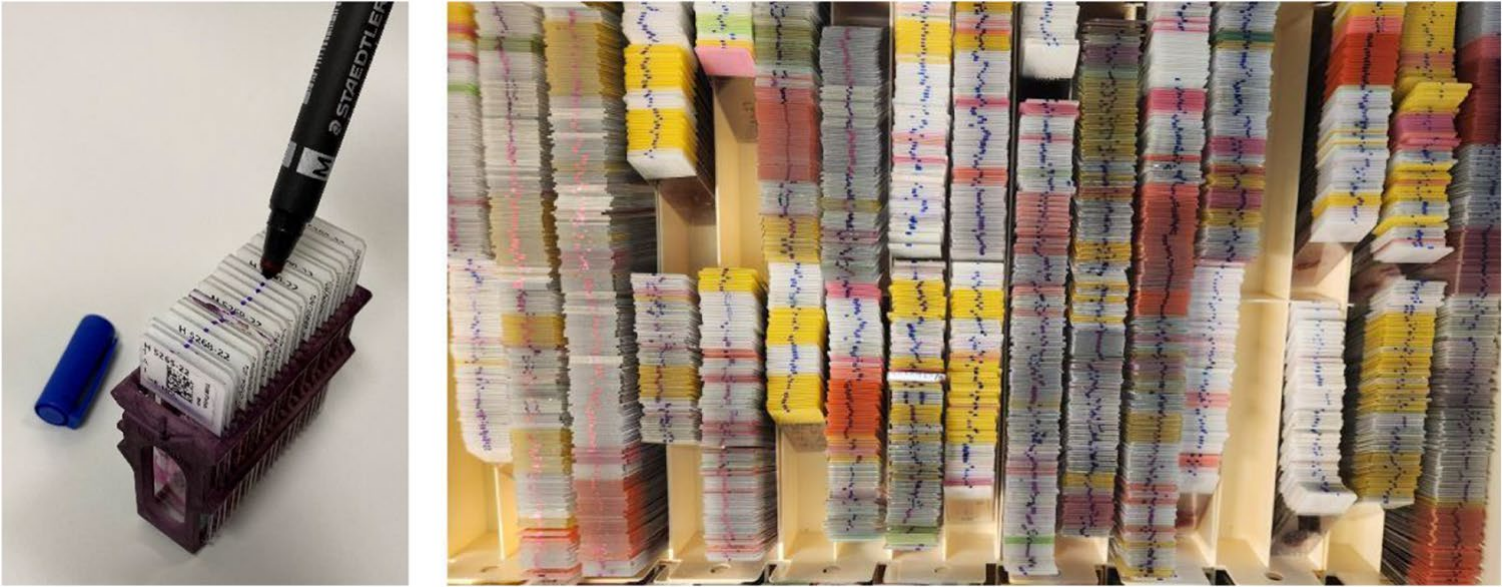
Left: digitalized slides are marked with a pen. Right: during the transition period, these markings enable the quick identification of scanned and unscanned slides

Within 1 year, all subgroups of histopathological samples were transitioned to prospective scanning, and all routine cases are now available *fully digitally at the same time as the glass slides.* Cytology slides and frozen sections are excluded from scanning, as the scanners are still too inaccurate or slow for those cases. The final setup includes two Glissando 20SL (Objective Imaging) for double-sized glass slides of prostate whole mounts and six Aperio GT450Dx for all other slides. Due to space limitations in the main laboratory, we accommodate a dedicated scanning room with customized lifting tables, each fitting two scanners vertically (Fig. 1). With this setup, we scan around 800–1000 routine slides daily. Digitalized slides are marked with a pen and kept in the archive (Fig. 2). These markings are helpful in the transition period to differentiate scanned from unscanned slides in the archive. Finally, the number of physically delivered slides has progressively been reduced. To ensure a seamless digital slide reviewing experience, each pathologist’s workplace was equipped with a second computer screen (27″, 2560 × 1440 QHD) and a 1 Gb/s LAN connection. Our institute uses the MSK/TUM slide viewer [30] that is being used at the Memorial Sloan Kettering Cancer Center (NY, USA) as a digital platform for primary diagnosis [4, 16, 20, 31].

## Validation study for digital workflow

While prospectively scanning more tissue groups, we have started the validation study for digital sign-out. The study’s objective was to provide a high degree of quality and confidence in our DP workflow while simultaneously quantifying any potential discrepancies. For this, we incorporated the guidelines of the College of American Pathologists (CAP) [24], the Royal College of Pathologists (RCPath) [32], and the Federal Association of German Pathologists (BDP) [33]. Following these guidelines, we implemented a two-phase approach.

**Fig. 3:**
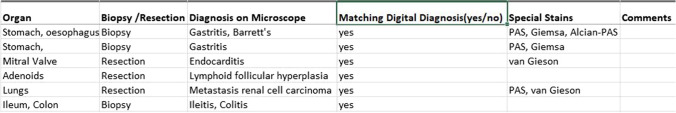
Example sheet for the collected feedback in the training phase

In the initial *training phase*, each pathologist and assistant pathologist reviewed 15 of their incoming cases digitally immediately after conventional sign-out. This phase aims to acquire experience on how diverse morphologic patterns reflect in a digital image and their feedback on the digital workflow. For this, the pathologists filled out a form to confirm whether or not the microscopic examination could be confirmed and if there were any feedback or ideas to improve the workflow (Fig. 3).

In the consecutive *validation phase*, each pathologist digitally reviewed 60 retrospective cases blinded to their original microscopic report. This phase aims to acquire more feedback on the digital workflow and to quantify potential discrepancies in the diagnostic process when using digital tools. For this, the study coordinator randomly selected cases for each pathologist that he or she signed out over 9 months before to maintain a wash-out period. The digital images of the selected cases were presented to the pathologists decoupled from the original case number and report but bundled with the clinical question and the macroscopic description to simulate the clinical workflow. Pathologists were then kindly asked to review the digital case and render a diagnostic report. They should skip cases they remember and review an additional case instead. Each pathologist was only presented with cases of their own worklist to exclude inter-pathologist variations in the diagnosis. Cases were selected from typical workdays to match a representative spectrum considering case size, difficulty, and diversity. The new diagnostic reports were then compared with the original pathology reports for evaluation. The digital case review was classified as *no discrepancy* (original and digital reports correspond to each other), *minor discrepancy* (original and digital reports differ in descriptions but not in the final diagnosis), or *major discrepancy* (original and digital reports differ in final diagnosis).

## Results

In the *training phase,* 14 pathologists (including assistants) reviewed a total of 210 cases. In this 3-month phase, we collected initial feedback for the digital sign-out workflow. Four pathologists noted discrepancies between their diagnoses and the digital images, accompanied by specific observations: (i) inability to detect H. Pylori in digital images (*n* = 2), (ii) need for polarization (*n* = 1), and (iii) challenge of discerning melanin pigment due to an oversaturation of colors by digital tools (*n* = 1). There were additional remarks highlighting difficulties in transitioning between magnification levels and navigating specimens efficiently, particularly in comparison to traditional microscopy. Other non-diagnostic comments were as follows:

- Setting the virtual HPF for mitotic counting to 0.16 mm.^2^
- The area at the edge of a slide with tissue was not covered with a coverslip
- When looking at the slide through a microscope, it is faster to navigate through the cut to find/exclude something (e.g., in searching for metastasis in a lymph node and other situations). Unfortunately, this is not so on digital because of the low frame rate. It could work better with a faster connection to the server.
- A correct diagnosis can be made quickly and easily, like with a microscope.
- DP generally works well as an alternative to the classical microscope, and it also offers some advantages, especially in the overview, such as better assessment of the sedimentation rings, simple precise measurements, and the possibility to view several different images simultaneously.

We addressed the concerns and enhanced our digital slide viewer with a new functionality to zoom slides faster, a pre-set size of an HPF, and optimizing the slide rendering viewer logic for faster visualization. Further, we optimized the slide storage so that digital slides remain on fast storage for 2 weeks before they are moved to the long-term network storage, thus minimizing network delays and improving the slide navigation experience.

**Fig. 4:**
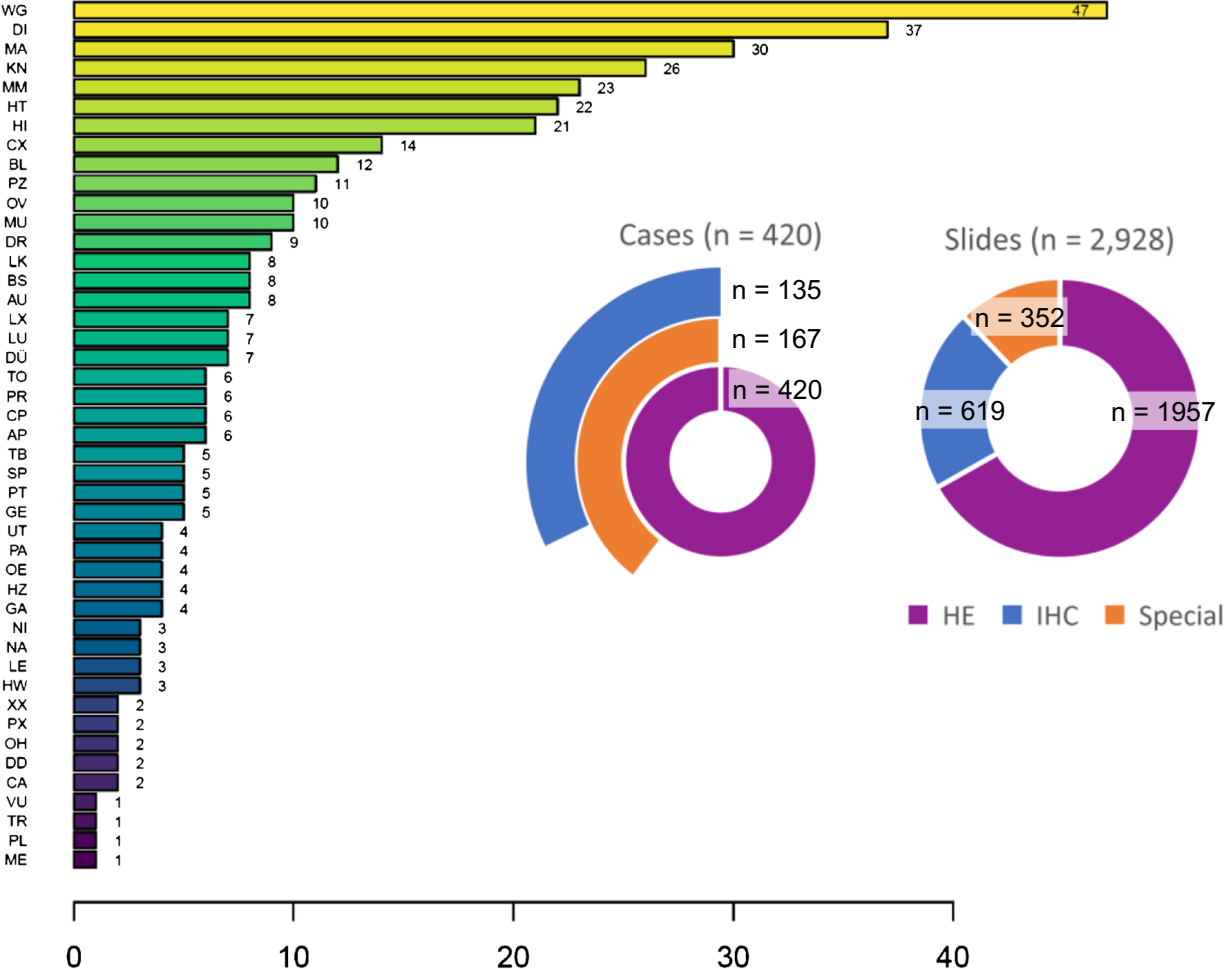
Tissue sites and staining protocols covered by our study. Left bar plot: The most prominent tissues are soft tissue (WG, *n* = 47), colon (DI, *n* = 37), and stomach (MA, *n* = 30). Right pie charts: The 420 cases of our study include special stains (*n* = 167) and IHC (*n* = 135). This translates to 1957 HE slides, 619 IHC slides, and 352 slides with special stains

In the *validation phase*, 7 expert pathologists reviewed a total of 420 cases. Case sizes ranged from 1 to 51 slides (mean 7 slides, median 4 slides) with a total of 2928 slides. A total of 135 (32%) of the cases included IHC staining (619 IHC slides of over 100 different antibodies). And 167 (40%) of the cases included special stains (352 slides of PAS, van Gieson, Giemsa, Iron, or others). Cases were distributed over 46 tissue sites, including common types such as the soft tissue (*n* = 47), colon (*n* = 37), or stomach (*n* = 30), and rare types such as vulva, trachea, pleurae, and mediastinum (each *n* = 1), ensuring a wide range of applications and staining protocols covered by our study (Fig. 4).

**Fig. 5:**
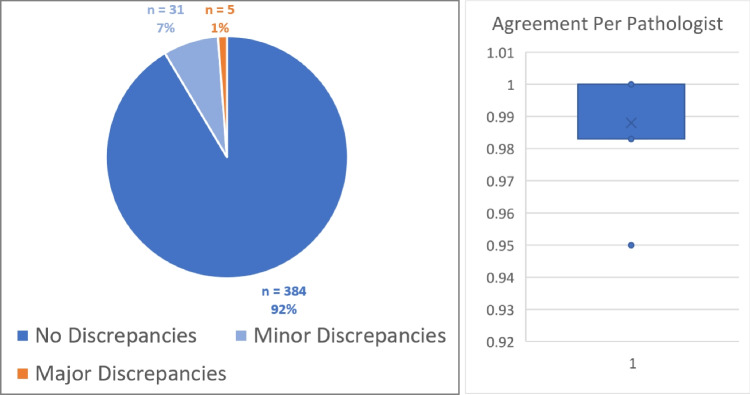
Results of the validation study: Left: 415 (98.8%) cases showed no discrepancies impacting the final diagnosis. For five cases (1.2%), the digital diagnosis differed from the manually rendered version. Right: The agreement (= no or minor discrepancy) per pathologist. Four pathologists showed 100% agreement, two showed 98.3% agreement, and one showed 95.0% agreement (median 100%)

The original report was typically more verbose and comprehensive than the new digital study report. In cases where the digital study report was not comprehensive enough to make a comparison, the corresponding pathologist was asked to complete the digital report, still blinded to the case. Of all 420 cases, 384 (91.4%) showed *no discrepancies* in the report, 31 (7.4%) showed *minor discrepancies*, and 5 (1.2%) showed *major discrepancies* leading to a change in the diagnosis. *Major discrepancies* were associated with borderline cases of HER2 estimation (close at the 0/1 + border) (*n* = 1) or non-detected inflammation in the original report (*n* = 1). Of all seven pathologists, four had no *major discrepancies*, two had one *major discrepancy*, and one had three *major discrepancies*, indicating an individual effect of DP rather than a systematic impact. Regarding scan quality, two scans were reported as blurry but without affecting the diagnosis. We define case *agreement* as the relative number of cases with *no discrepancy* or *minor discrepancy*. Our study thus results in 98.8% agreement between all digital and analog case reviews, with a median agreement of 100% among all pathologists and a minimal agreement of 95.0% for one pathologist (Fig. 5).

## Efficiency of digital case review

**Fig. 6:**
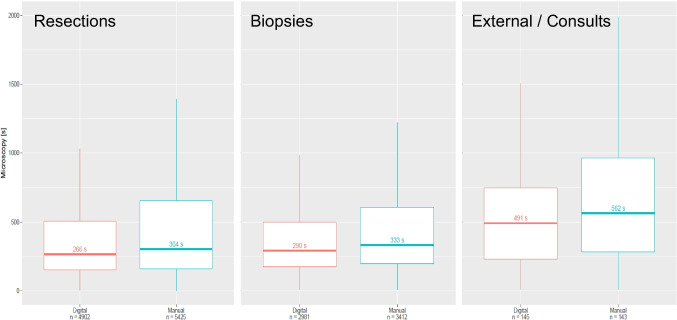
Time differences between digital and manual case review of resections (left, *n* = 4902/5425), biopsies (middle, *n* = 2981/3412), and external cases (right, *n* = 145/143). Shown is the median time for opening the case and closing it for sign-out by a pathologist. Digital case review is slightly faster in all three scenarios, with a median time saving of 38 s, 43 s, and 71 s, respectively

DP fundamentally changes the way pathologists review cases. While the interaction with a computer mouse to navigate a slide is commonly perceived as less efficient than using a microscope, the search for local image spots on a digital overview image, the retrieval or prior cases of a patient, and the change of slides within a case are sped up with DP. The resulting overall impact on efficiency is unclear. Therefore, we measured the time to sign out cases before and after the transition to DP. For this, we compared cases from 6 months before digital transitions with cases of the same time range after digital transition, thus accounting for approximately similar case numbers and distribution. For each case, the time between opening the case by a certified pathologist and closing the case, both recorded in the LIS, was measured. To account for differences between *larger* and *smaller* cases, we stratified the results by resections and biopsies. Further, we considered external cases as a special group as they come with varying sizes and potentially require further assessments. Throughout all cases, we found a decrease in review time after our digital transition, as detailed in Fig. 6. From 10,327 included resections, 5425 cases have been signed out analogously with a median time of 304 s, and 4902 cases have been signed out digitally with a median time of 266 s (− 38 s). Similarly, from 6393 included biopsies, 3412 have been signed out analogously with a median time of 333 s, and 2981 have been signed out digitally with a median time of 290 s (− 43 s). Finally, from 288 included external cases, 143 have been signed out analogously with a median time of 562 s, and 145 have been signed out digitally with a median time of 491 s (− 71 s).

## Quality control and error handling

In 2024, our institute was accredited according to the DIN EN ISO/IEC 17020, including DP. This requires an appropriate quality management system to be able to identify problems with the digital workflow in a timely manner, keeping a record of scanner issues and digital slide problems. Besides the diagnostic-grade scanners’ internal quality control measures, the scanning team systematically reviews approximately 10% of the digital slides (two slides per rack) after scanning. When artifacts such as blurriness occur, a rescan is triggered. Pathologists can further report low-quality slides via the viewing software’s report function to the scanning team such that a rescan and/or physical delivery of the glass slides is triggered. As slide rescanning should be quick, we see a large potential for viewing software that can directly interact with the scanner so that a pathologist can rescan any slide without delay. In 1 month of clinical routine, we scanned 20,635 slides, of which 29 (1.4‰) were reported by the pathologists for low quality and needed to be rescanned. Eighteen (0.9‰) slides have been ordered for delivery for multiple reasons: scan out of focus (*n* = 10), tissue out of scanning area (*n* = 3), required polarization (*n* = 3), missing coverslip (*n* = 1), or tiny tissue (*n* = 1). We noted that in rare cases, very small tissues or very pale stains can be problematic for the tissue finder of the scanners and therefore cannot be scanned. As such, in-house validation quality assurance program (QAP) during DP implementation, and continuous quality control (QC) activities, including pre- and post-scan QCs are essential for assuring the quality of pathologic practice in each laboratory [34, 35].

## Our implementation strategy using existing guidelines

To ensure reliability and accuracy in DP practices, leading pathology organizations such as the Royal College of Pathologists (RCPath) [32], the College of American Pathologists (CAP) [24], and the Federal Association of German Pathologists (BDP) [33] have developed validation guidelines. Based on these guidelines, we followed a two-phase validation process tailored to our institution’s unique needs.

### Training phase

We conducted an initial validation phase based on CAP’s suggested case volume (60 cases). We included 210 cases, focusing on primary diagnostic accuracy and workflow efficiency. This phase helped identify immediate integration challenges and highlighted areas requiring optimization.

### Validation phase

Using RCPath’s recommendation for a broader case volume, we expanded validation to ensure system robustness across different tissue types and diagnostic complexities. The RCPath guidelines on environmental requirements were particularly beneficial, leading us to establish dedicated digital diagnostic workstations with high-resolution monitors and color calibration.

### Segmented/modular and risk-based validation

We adopted the BDP’s modular approach to validate specific DP components (e.g., telepathology tools) and to revalidate the system as software updates or new functionalities were introduced. This flexible, risk-based validation strategy allowed for a more efficient process while maintaining diagnostic accuracy.

### Benefits and challenges in implementation

The combination of guidelines allowed us to develop a comprehensive and scalable DP system. Key benefits are as follows:

- Enhanced diagnostic accuracy and concordance: with RCPath’s emphasis on thorough validation, we achieved high diagnostic accuracy, aligning digital and traditional results in over 98% of cases.
- Resource and time efficiency: CAP’s streamlined caseload recommendations provided a baseline, minimizing initial resource demands and expediting the adoption process. BDP’s phased approach further optimized resource use by tailoring validation to clinical relevance.
- Integrating DP within established workflows required extensive training and adaptation, which we addressed with CAP’s guidance on user training and workflow integration.

The integration of DP is a complex endeavor that requires thorough validation to ensure reliability and accuracy. By synthesizing the guidelines from RCPath, CAP, and BDP, we developed a robust, validated DP workflow tailored to our institution’s unique needs. Each set of guidelines contributed essential insights, allowing us to create a comprehensive, flexible, and compliant DP system that supports both diagnostic excellence and regulatory compliance. The balanced approach between rigor and practicality afforded by these guidelines facilitated a smooth transition to digital workflows, setting a solid foundation for future innovations in DP.

## Discussion

We conducted a two-phase approach with a learning phase and a validation phase to adopt DP. Pathologists could provide feedback in each phase and were deeply involved in our transition, e.g., by identifying case groups that benefit most (biopsies, IHC) or least (cytology, polarization) from digital review. This strongly fostered acceptance and adoption of DP.

We decided to include all pathologists in the first phase of the study, including assistants, to gather as much feedback as possible since they will all use DP in the future. Still, we excluded assistants’ results in the validation phase for two reasons: First, assistants do not render their own (manual) diagnosis needed to compare to, and second, any discrepancies between original and digital reports would not necessarily be attributable to the digital workflow (or to their early training status).

We ran the validation phase with 60 cases per pathologist, collecting those samples from their retrospective worklists. This data set may not capture the full range of challenges and nuances encountered over a whole year. However, the study’s main aim is to familiarize pathologists with a digital workflow and to make them aware of potential differences in the two modalities, enabling them to recognize (new) cases in which they might be unsure. We recommend to our future pathologists who use DP to fulfill a similar study protocol to get a controlled feeling digital slide review.

This study focuses solely on the diagnostic alignment between digital and microscopic methods without exploring other potential factors influencing diagnostic accuracy, such as variations in slide preparation techniques. This limitation may restrict the generalizability of the findings and warrant caution in extrapolating the results to broader contexts within clinical practice. Therefore, every transitioning lab must run its own validation study.

The validation process yielded valuable insights into the effectiveness of the transition to DP. By involving a diverse group of pathologists, including experienced practitioners and assistant pathologists, the study captured a broad spectrum of perspectives and experiences. During both the training and study phases, pathologists provided comments, suggestions, and opinions, highlighting areas of improvement and potential challenges in adopting DP. These insights were instrumental in refining the digital workflow and addressing concerns raised by pathologists, ultimately enhancing the usability and acceptance of DP systems. The re-evaluation of old cases allowed for a comprehensive assessment of the reliability and accuracy of DP compared with manual microscopy. Pathologists’ ability to accurately interpret and diagnose cases digitally, without bias from prior manual evaluations, demonstrated the robustness of the digital platform and its potential to streamline diagnostic workflows. Our pathologists achieved an agreement in digital and manual slide reviews in 98.8% of the cases. Four pathologists agreed in 100%, two pathologists in 98.3%, and one pathologist in 95% of their cases. This result suggests that the impact of DP on slide review depends on the individual expert.

A major concern when implementing DP is TAT. While there is limited literature on the effect of DP on TAT, the results of such studies vary from generally decreased, decreased limited to specific specimens, to no change and increased TAT [37–41]. Our study implies that DP has the potential to decrease TAT, but this potential can only be realized when appropriate implementation is achieved, including all aspects of a high-quality DP workflow.

Rigorous regular maintenance of the scanners is crucial to ensuring consistent performance. The scanners are highly sensitive to dust, dirt, wax, or glue residues, easily leading to scanner errors or poor image quality (stripe artifacts or others). Furthermore, environmental factors, such as excessive heat in summer, also impacted the overall scanning operation. We therefore installed an air conditioning unknit in the scanning room.

We implemented a pre-scan quality check assessing each slide’s physical condition before scanning. This includes a check whether the slide is wet, which could damage the scanner or lead to poor-quality images, whether the barcode is clearly legible, and whether the coverslip is present and properly aligned. Protruding coverslips put the scanner lenses at risk of damage.

We aim to keep our institute’s *same-day* scanning policy. However, on exceptional days with a high workload (too many slides or too few laboratory staff due to illness), it is necessary to adapt the workflow by outsourcing individual cases that are then issued to the doctors and scanned afterward.

## Conclusion

In our study, digital case review was equivalent to manual case review regarding diagnostic findings. DP reduced case review times for resections, biopsies, and external cases despite initial concerns about its perceived inefficiency compared to traditional microscopy. These findings suggest that DP offers long-term efficiency benefits, making it a promising tool for modernizing pathology workflows.

The two-phase validation approach presented in this study offers a systematic and thorough method for assessing the transition of pathologists to DP. By incorporating feedback from participants and re-evaluating old cases, the study provides valuable insights into the efficacy and reliability of DP systems in routine practice.

As an institute, we have achieved significant advancements in integrating DP into our clinical diagnostic processes. Our pathologists now finalize routine case analyses utilizing DP. This transition has afforded our pathologists greater flexibility in their work schedules, including the option to work remotely.

Ongoing validation efforts and refinement of DP workflows are essential to ensure optimal performance and widespread adoption in clinical settings. Collaborative efforts between pathologists, laboratory staff, IT specialists, and healthcare institutions will play a crucial role in driving the successful adoption of DP into modern healthcare practice. Having unwavering leadership support with vision and commitment to resources is crucial to a successful DP.
